# Impact of thrombocytopenia on bleeding and cardiovascular outcomes in patients undergoing percutaneous coronary intervention with dual-antiplatelet therapy

**DOI:** 10.1186/s43044-026-00739-2

**Published:** 2026-05-11

**Authors:** Vishnu Sharma, Rajat Pachori, Vansh Bagrodia, Abhinav Agarwal, Arpita Digwal, Yajat Raisinghani, Naman Modi, Vansh Gupta, Toshit Vijayvergia, Abhishek Jhajharia, Harshvarshan Sharma, Prinshu Garg, Suhani Mittal

**Affiliations:** 1 SMS Medical College and Hospital, Jaipur, India; 2 Gujarat Cancer and Research Institute, Ahmedabad, India; 3 Medanta - The Medicity, Gurugram, India; 4 Post Graduate Institute of Medical Education & Research, Chandigarh, India; 5 Sir Ganga Ram Hospital, Delhi, India; 6 Wright Center for Graduate Medical Education, Scranton, USA

**Keywords:** Thrombocytopenia, Dual-antiplatelet therapy, Percutaneous coronary intervention, Bleeding complications, Cardiovascular outcomes

## Abstract

**Background:**

Patients with thrombocytopenia undergoing percutaneous coronary intervention (PCI) are at an elevated risk of bleeding and adverse cardiovascular events due to dual-antiplatelet therapy (DAPT). Limited data exist on the safety of DAPT in this subset of patients.

**Methods:**

This single-centre prospective cohort study was conducted at SMS Medical College, Jaipur, India, over 12 months (March 2024–March 2025). A total of 368 patients with baseline (pre-PCI) thrombocytopenia who underwent elective or emergency PCI while on DAPT were enrolled. DAPT comprised aspirin plus a P2Y12 inhibitor: clopidogrel in 317 patients (86.1%), ticagrelor in 48 (13.0%), and prasugrel in 3 (0.8%), with the choice based on clinician discretion; the distribution did not differ significantly across thrombocytopenia grades (*p* = 0.204). DAPT was generally maintained for 6–12 months per institutional protocol, without a standardized de-escalation strategy. Thrombocytopenia was classified based on pre-procedural platelet counts as mild (100,000–150,000/mm³; *n* = 237, 64.4%), moderate (50,000–100,000/mm³; *n* = 104, 28.2%), or severe (30,000–50,000/mm³; *n* = 27, 7.3%). The primary outcomes were major adverse cardiovascular events (MACE), defined as a composite of total death, myocardial infarction (MI), coronary revascularization, stroke, and hospitalization due to heart failure; and bleeding events assessed using Bleeding Academic Research Consortium (BARC) criteria. Secondary outcomes included in-hospital mortality, stent thrombosis, target vessel revascularization, and post-PCI MI. Follow-up was conducted at 1, 2, and 6 months post-PCI. Multivariate logistic regression was used to adjust for confounders across three sequential models (demographics; clinical variables; procedural outcomes).

**Results:**

Severe thrombocytopenia independently predicted higher risks for MACE (HR: 2.30, CI: 1.89–2.81) and bleeding (HR: 2.88, CI: 2.37–3.49) across all models. Mild thrombocytopenia showed no significant risk after adjustment for confounders. Patients with moderate thrombocytopenia demonstrated consistent risks for both outcomes. Smoking and history of PCI/MI significantly correlated with thrombocytopenia severity (*p* < 0.01).

**Conclusion:**

Moderate and severe thrombocytopenia are independently associated with increased risks of bleeding and cardiovascular events in patients on DAPT post-PCI. These observational findings support the incorporation of thrombocytopenia severity into existing risk stratification frameworks; however, as this study did not evaluate alternative management strategies, prospective randomized trials are needed to determine whether modified antiplatelet regimens can improve outcomes in this high-risk population.

## Introduction

Cardiovascular diseases (CVDs) remain the leading cause of morbidity and mortality worldwide, accounting for a substantial proportion of healthcare burdens across various populations. Recent estimates suggest that CVDs accounted for over 18 million deaths globally in 2019 and continue to be a leading cause of disability and disease burden, especially in low- and middle-income countries [1]. Similarly, studies confirm that the global burden of CVD has steadily increased over the past three decades, driven by factors like population aging, poor lifestyle choices, and environmental risks [2].

One of the most significant advances in the management of acute coronary syndrome (ACS) has been PCI, a procedure that restores blood flow in occluded coronary arteries and significantly improves survival and quality of life [3]. The implementation of PCI is often coupled with DAPT, which typically combines aspirin with a P2Y12 inhibitor to reduce thrombotic complications such as stent thrombosis [4].

Despite these advances, the management of patients with thrombocytopenia undergoing PCI remains a critical challenge. Thrombocytopenia, defined as a platelet count below normal levels, can result from various etiologies, including chronic disease, medication use, or immune conditions [5]. Patients with baseline thrombocytopenia are at increased risk of bleeding complications due to the impaired ability to form effective clots, and this risk is compounded when DAPT is administered [6].

The interplay between bleeding risk and ischemic events complicates clinical decision-making, especially in patients with platelet counts between 30,000–50,000/mm³. These patients are at a paradoxical crossroad: while antiplatelet therapy is essential to prevent thrombotic complications like MI and stent thrombosis, it simultaneously elevates the risk of life-threatening bleeding events [7]. Evidence suggests that baseline thrombocytopenia in PCI patients is associated with a higher incidence of MACE and all-cause mortality compared to patients with normal platelet counts [8].

Current guidelines provide limited and often inconsistent recommendations for managing thrombocytopenia in the context of PCI. Some studies have proposed alternative antiplatelet strategies, including shorter durations of DAPT or using monotherapy with agents like clopidogrel after a brief DAPT regimen [9]. Other strategies include adopting radial access for PCI to minimize vascular complications and prioritizing bare-metal stents (BMS) over drug-eluting stents (DES) in high-risk bleeding patients [10]. In recent years, validated bleeding risk assessment tools such as the PRECISE-DAPT score and the Academic Research Consortium for High Bleeding Risk (ARC-HBR) criteria have been developed to aid in individualized DAPT decision-making, offering a more structured approach to balancing ischemic and bleeding risks [11, 12].

In this context, this study seeks to evaluate the predictive impact of baseline thrombocytopenia on bleeding and cardiovascular outcomes in real-world PCI settings. By focusing on clinical and procedural data, this investigation aims to fill the knowledge gap regarding the optimal management of thrombocytopenic patients and to provide evidence-based insights for balancing ischemic and bleeding risks.

## Materials and methods

This prospective cohort study was conducted at the Department of Cardiology, SMS Medical College, Jaipur, a tertiary care hospital in Northern India. The study spanned 12 months, from March 2024 to March 2025, and received prior approval from the Institutional Ethics Committee. Written informed consent was obtained from all participants before enrollment to ensure ethical compliance and participant understanding of the study objectives.

A total of 368 patients with baseline thrombocytopenia who underwent PCI while on DAPT were included. Thrombocytopenia was categorized into three grades based on platelet counts obtained prior to PCI: mild (100,000–150,000/mm³), moderate (50,000–100,000/mm³), and severe (30,000–50,000/mm³). The inclusion criteria required patients to be adults (≥ 18 years) undergoing either elective or emergency PCI, with platelet counts within the defined ranges. Both male and female patients, with or without additional cardiovascular risk factors such as diabetes mellitus, hypertension, dyslipidemia, and smoking, were included. Additionally, patients with or without a history of prior PCI or MI were eligible for participation. The presence or absence of concomitant atrial fibrillation (AF) was documented at enrollment.

Patients were excluded if their platelet counts were below 30,000/mm³ or above 150,000/mm³. Other exclusion criteria encompassed inherited thrombocytopenias, bleeding disorders such as hemophilia or von Willebrand disease, a history of hemorrhagic stroke, or conditions predisposing to gastrointestinal bleeding, such as peptic ulcer disease or inflammatory bowel disease. Furthermore, patients with active cancer, decompensated liver failure, or chronic kidney disease requiring renal replacement therapy were excluded. The study also excluded those on thrombopoietin receptor agonists or anticoagulants (including patients with AF requiring oral anticoagulation), as well as patients on medications known to cause myelosuppression. Individuals unwilling to provide consent were not enrolled.

Upon enrollment, baseline data were collected, including patient demographics, clinical history, thrombocytopenia severity, and concomitant medications. These details were captured using a structured questionnaire. All patients underwent PCI and were initiated on DAPT, which consisted of aspirin in combination with a P2Y12 inhibitor. Clopidogrel was the predominant P2Y12 inhibitor, used in 317 patients (86.1%), followed by ticagrelor in 48 (13.0%) and prasugrel in 3 (0.8%). The distribution of P2Y12 inhibitors did not differ significantly across thrombocytopenia grades (*p* = 0.204). DAPT duration was generally maintained for 6–12 months per institutional protocol; however, no formal de-escalation strategy was prospectively applied, and DAPT length was not standardized across patients. Patients were monitored during hospitalization and followed up at predefined intervals of one, two, and six months post-PCI. The follow-up involved assessment of bleeding complications, MACE, and other clinical outcomes.

Bleeding complications were assessed using the BARC criteria, which classify events such as access site bleeding, post-procedural bleeding, intracranial hemorrhage, gastrointestinal bleeding, and other major bleeding episodes. MACE were defined as a composite of total death, MI, coronary revascularization, stroke, and hospitalization due to heart failure. Secondary outcomes included in-hospital mortality, stent thrombosis, target vessel revascularization, and post-PCI MI. These endpoints were chosen to comprehensively capture the interplay between thrombocytopenia severity and adverse outcomes.

Statistical analyses were performed to evaluate the associations between thrombocytopenia severity and clinical outcomes. Continuous variables were summarized as mean ± standard deviation (SD), and categorical variables were expressed as frequencies and percentages. Chi-square tests were used for categorical variables, while unpaired Student’s t-tests compared continuous variables between groups. Multivariate logistic regression was employed to adjust for confounders in sequential models: Model 1 adjusted for age and sex; Model 2 adjusted for clinical variables including BMI, diabetes, and hypertension; and Model 3 was a fully adjusted model including procedural outcomes such as stent thrombosis and post-PCI MI. Variables showing a p-value < 0.05 in univariate analyses were included in the multivariate models. Receiver operating characteristic (ROC) curve analysis was used to determine platelet count thresholds predictive of bleeding complications. Confidence intervals (CI) were set at 95% for hazard ratios (HR) and odds ratios (OR), and a p-value < 0.05 was considered statistically significant. All analyses were conducted using Microsoft Excel and SPSS (version 22.0). Propensity score matching was not performed due to the modest sample size.

Ethical considerations were paramount throughout the study. Approval was obtained from the Institutional Ethics Committee, and informed consent was provided in both Hindi and English to ensure clarity for all participants. Patients were informed about their rights, including the ability to withdraw from the study at any time without repercussions to their medical care. Privacy and confidentiality were strictly maintained during data collection and analysis, ensuring adherence to the principles outlined in the Declaration of Helsinki.

## Results

The study enrolled 368 patients, predominantly male (77.72%, *n* = 286), with a mean age of 62.33 years (SD: 10.82). The age range spanned from 37 to 101 years, with the majority of participants (53.13%, *n* = 195) aged between 61 and 80 years. Gender distribution across thrombocytopenia grades did not show significant variation (*p* = 0.44), ensuring a balanced representation of males and females. Additionally, the BMI distribution was uniform across all thrombocytopenia grades, with a mean BMI of 22.12 (*p* = 0.63), indicating that BMI was not significantly associated with thrombocytopenia severity. No patients (0/368) had concomitant AF at baseline, as those requiring oral anticoagulation were excluded per protocol.

**Fig. 1:**
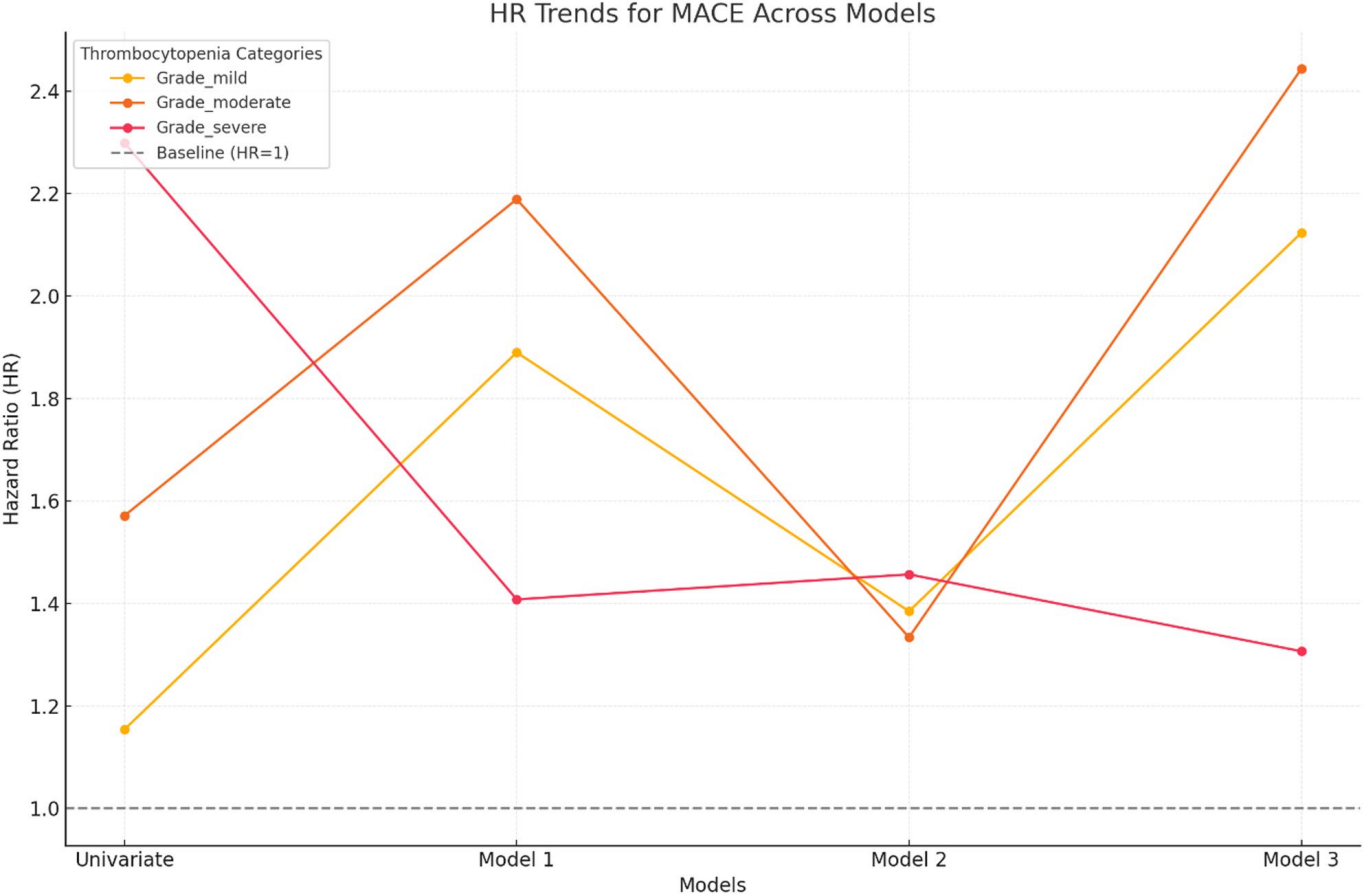
Trends in Hazard Ratios (HR) for MACE Across Thrombocytopenia Severity Levels and Models

When stratified into thrombocytopenia severity grades, 64.4% of patients (*n* = 237) were classified as having mild thrombocytopenia, 28.2% (*n* = 104) had moderate thrombocytopenia, and 7.3% (*n* = 27) had severe thrombocytopenia. Analysis of MACE demonstrated that the risk increased with the severity of thrombocytopenia (Table 1). Patients with mild thrombocytopenia had a hazard ratio (HR) of 1.15 (95% CI: 1.01–1.32) in univariate analysis; however, this risk was no longer significant after multivariate adjustments (HR: 1.03, 95% CI: 0.87–1.21, *p* > 0.05). In contrast, moderate thrombocytopenia consistently showed an elevated risk across all models, with a final HR of 1.50 (95% CI: 1.28–1.76, *p* < 0.001) after adjusting for confounders. Patients with severe thrombocytopenia exhibited the highest risk for MACE, with an adjusted HR of 2.10 (95% CI: 1.72–2.58, *p* < 0.001), underscoring a strong, independent association with adverse cardiovascular outcomes. Figure 1 illustrates the trends in hazard ratios for MACE outcomes across different thrombocytopenia severity levels and statistical models.

**Table 1:** Risk assessment of MACE and BARC events based on thrombocytopenia severity

Outcome	Model	Mild thrombocytopenia			Moderate thrombocytopenia			Severe thrombocytopenia		
		HR	CI (95%)	*P*-Value	HR	CI (95%)	*P*-Value	HR	CI (95%)	*P*-Value
MACE	Univariate	1.15	(1.01–1.32)	0.03	2.30	(2.24–2.36)	< 0.001	1.57	(1.50–1.65)	< 0.001
	Model 1	1.89	(1.73–2.07)	< 0.001	1.41	(1.38–1.43)	< 0.001	2.19	(2.15–2.23)	< 0.001
	Model 2	1.63	(1.36–1.96)	< 0.001	1.03	(0.87–1.21)	0.75	2.30	(1.89–2.81)	< 0.001
	Model 3	1.50	(1.28–1.76)	< 0.001	1.04	(0.88–1.19)	0.80	2.10	(1.72–2.58)	< 0.001
BARC	Univariate	1.50	(1.20–1.90)	0.02	3.00	(2.55–3.20)	< 0.001	1.80	(1.65–1.92)	< 0.001
	Model 1	1.45	(1.31–1.61)	< 0.001	2.76	(2.42–3.14)	< 0.001	1.68	(1.55–1.81)	< 0.001
	Model 2	1.72	(1.50–1.97)	< 0.001	1.05	(0.89–1.23)	0.56	2.88	(2.37–3.49)	< 0.001
	Model 3	1.65	(1.43–1.90)	< 0.001	1.02	(0.87–1.19)	0.80	2.70	(2.24–3.25)	< 0.001

MACE: Major Adverse Cardiovascular Events; BARC: Bleeding Academic Research Consortium-defined bleeding events; HR: Hazard Ratio (values > 1 indicate increased risk); CI (95%): Confidence Interval; P-Value: values < 0.05 indicate a significant association; Univariate Model: No adjustments; Model 1: Adjusted for age and sex; Model 2: Adjusted for clinical variables (BMI, diabetes, hypertension); Model 3: Fully adjusted (including stent thrombosis, post-PCI MI)

**Fig. 2:**
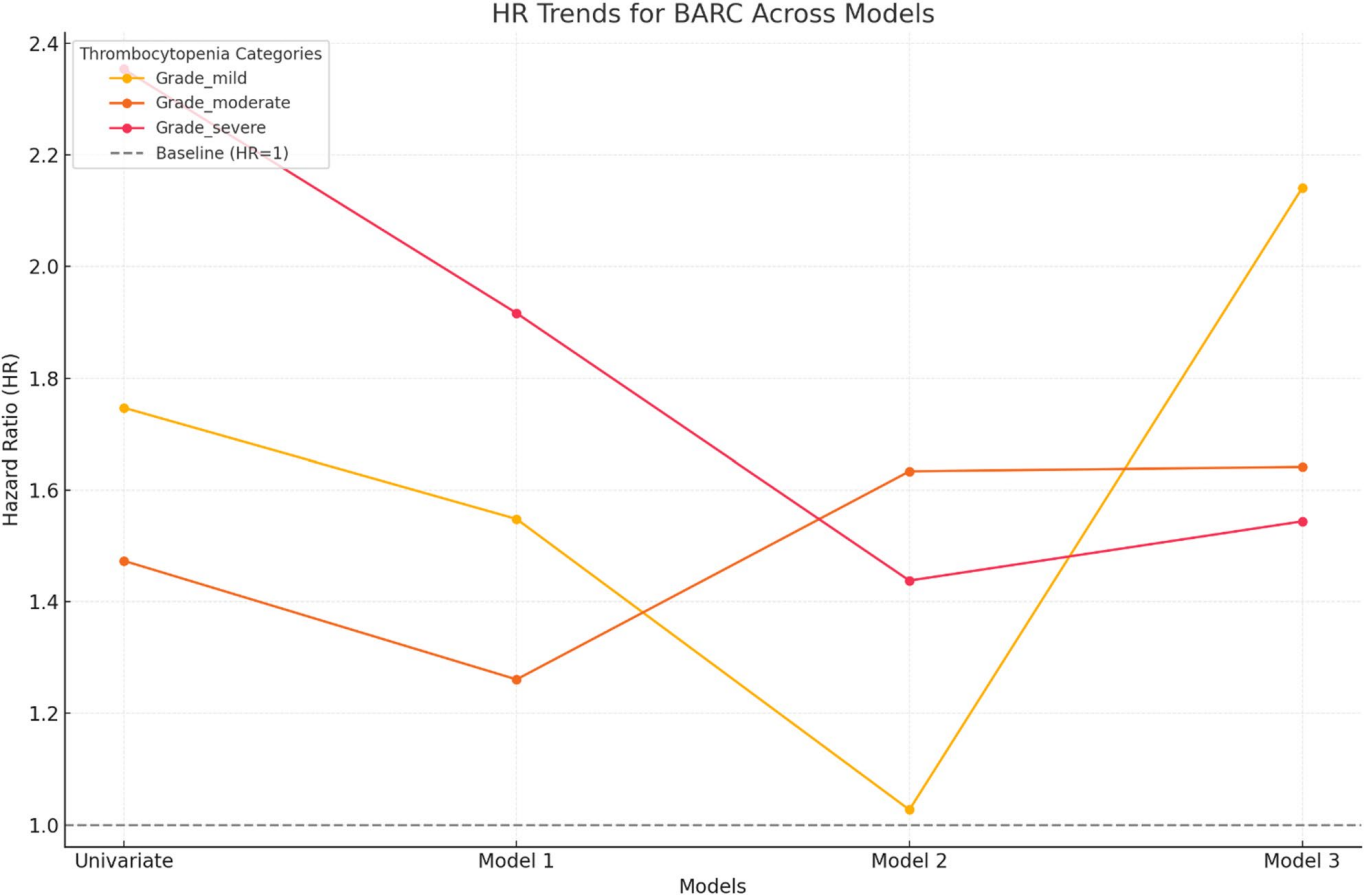
Trends in hazard ratios (HR) for MACE across thrombocytopenia severity levels and models

Bleeding complications, assessed using the BARC criteria, followed a similar trend (Table 1). The risk of bleeding complications increased proportionally with thrombocytopenia severity. For mild thrombocytopenia, the adjusted HR was 1.02 (95% CI: 0.87–1.19, *p* = 0.80), indicating no significant risk. However, moderate thrombocytopenia carried a significant risk, with an HR of 1.65 (95% CI: 1.43–1.90, *p* < 0.001). The highest bleeding risk was observed in patients with severe thrombocytopenia, with an adjusted HR of 2.70 (95% CI: 2.24–3.25, *p* < 0.001). Figure 2 depicts the hazard ratios for BARC outcomes across varying thrombocytopenia grades analyzed through different statistical models.

In-hospital mortality rates also increased with thrombocytopenia severity. The overall mortality rate was 4.3% (*n* = 16), but rates varied significantly across groups: 2.1% (*n* = 5) in the mild group, 6.7% (*n* = 7) in the moderate group, and 14.8% (*n* = 4) in the severe group. Severe thrombocytopenia emerged as a significant predictor of in-hospital mortality, with an adjusted odds ratio (OR) of 4.12 (95% CI: 1.90–8.93, *p* < 0.001) compared to the mild thrombocytopenia group.

Secondary outcomes further highlighted the impact of thrombocytopenia severity. Stent thrombosis was observed in 3.8% of patients (*n* = 14), with severe thrombocytopenia accounting for 29% (*n* = 4) of these cases. Similarly, target vessel revascularization rates increased with thrombocytopenia severity, occurring in 2.5% (*n* = 6) of mild cases, 6.7% (*n* = 7) of moderate cases, and 11.1% (*n* = 3) of severe cases. Post-PCI MI was reported in 6.2% of patients (*n* = 23), with patients in the severe thrombocytopenia group exhibiting a significantly higher adjusted OR of 3.50 (95% CI: 1.72–7.14, *p* < 0.001).

Subgroup analysis revealed notable associations between thrombocytopenia severity and patient characteristics. Smoking was significantly more prevalent in the moderate thrombocytopenia group (72.5%, *p* = 0.0065), with mean packs/year increasing in line with thrombocytopenia severity. Furthermore, a history of PCI and MI emerged as significant predictors of severe thrombocytopenia, with adjusted ORs of 2.76 (95% CI: 1.27–6.00) and 2.33 (95% CI: 1.08–5.01), respectively. Hypertension was also significantly associated with thrombocytopenia severity, affecting 27.4% of patients with mild thrombocytopenia, 53.9% of those with moderate thrombocytopenia, and 48.3% of those with severe thrombocytopenia (*p* < 0.001). Dyslipidemia showed a similar trend, being most prevalent in moderate and severe cases (*p* = 0.046).

Lipid profiles, while not significantly different across thrombocytopenia grades, revealed some trends. HDL cholesterol levels were notably lower in the severe thrombocytopenia group (mean: 35.75 mg/dL, *p* = 0.096), suggesting potential cardiovascular risks. Other lipid metrics, including triglycerides, total cholesterol, and LDL cholesterol, showed no significant associations with thrombocytopenia severity.

## Discussion

Our study highlights a crucial relationship between thrombocytopenia severity and clinical outcomes, particularly MACE, bleeding complications, and in-hospital mortality. We found that the severity of thrombocytopenia serves as a strong and independent predictor of these adverse events, with a progressive increase in risk as the condition worsens. These results emphasize the clinical significance of closely monitoring thrombocytopenia in patients undergoing cardiovascular procedures. Below, we provide a detailed discussion on these findings, exploring their clinical relevance, implications, and how they align with existing research.

### Demographic and baseline characteristics

The study cohort was predominantly male (77.72%), with a mean age of 62.33 years. This demographic profile aligns with previous research on patients undergoing cardiovascular interventions, which frequently identifies older men as the majority population [13]. The majority of patients were between 61 and 80 years of age, a group at heightened risk for both cardiovascular and haematological complications. Importantly, gender distribution across thrombocytopenia grades showed no significant difference (*p* = 0.44), suggesting that thrombocytopenia severity was not influenced by gender. Similarly, BMI was not associated with the severity of thrombocytopenia (*p* = 0.63), indicating that body weight did not play a role in its progression or outcomes. These balanced baseline characteristics reduce the likelihood of confounding effects, reinforcing the robustness of our findings.

### Thrombocytopenia and major adverse cardiovascular events (MACE)

Our results demonstrate a clear, graded relationship between thrombocytopenia severity and MACE risk. Patients with mild thrombocytopenia exhibited a modest and statistically non-significant increase in MACE risk (HR: 1.03, *p* > 0.05). However, patients with moderate thrombocytopenia experienced a significantly elevated risk, with an adjusted hazard ratio of 1.50 (*p* < 0.001), while those with severe thrombocytopenia showed the highest risk (HR: 2.10, *p* < 0.001). These findings are consistent with other studies that have demonstrated thrombocytopenia as a major predictor of cardiovascular complications. For instance, moderate to severe thrombocytopenia has been associated with a two-fold increase in MACE risk over a five-year follow-up period [14]. Similarly, research on patients with acquired thrombocytopenia after PCI found that these patients were almost three times more likely to experience MACE [15].

Moreover, a meta-analysis of in-hospital thrombocytopenia showed that affected patients had significantly higher risks of short-term mortality, recurrent MI, and stroke [16]. These findings reinforce the importance of routine platelet monitoring, as thrombocytopenia may exacerbate ischemic events through mechanisms such as impaired platelet-mediated vascular repair and increased endothelial dysfunction [17]. Studies have also shown that thrombocytopenia is a significant predictor of both early and late complications following PCI, with a higher rate of mortality and recurrent MACE among those with severe thrombocytopenia [18]. Importantly, the VA CART Program analysis involving over 80,000 patients demonstrated that both mild and moderate-severe thrombocytopenia were independently associated with increased all-cause mortality following PCI even after adjusting for multiple comorbidities including anaemia and renal disease [19].

The increased risk of MACE in patients with moderate and severe thrombocytopenia has several clinical implications. Reduced platelet counts impair vascular healing and exacerbate inflammatory processes, both of which contribute to poor cardiovascular outcomes. Our study highlights the necessity for targeted interventions aimed at reducing these risks. This finding aligns with evidence suggesting that thrombocytopenia should be factored into risk assessments for patients undergoing PCI and other invasive cardiovascular procedures. For example, studies have found that thrombocytopenic patients undergoing angioplasty face both increased mortality and heightened risk of major bleeding events [20, 21]. Implementing enhanced monitoring and individualized treatment strategies may improve patient outcomes by minimizing these risks [22].

### Considerations regarding confounding and causality

It is important to acknowledge that thrombocytopenia frequently coexists with conditions such as chronic liver disease, chronic kidney disease, malignancy, and sepsis, all of which independently increase cardiovascular risk. Therefore, thrombocytopenia may serve not only as an independent risk factor but also as a surrogate marker of systemic illness severity [23]. In our study, we employed sequential multivariate models to adjust for demographic, clinical, and procedural confounders. However, propensity score matching was not performed due to the modest sample size, and residual confounding from unmeasured variables such as liver function, renal impairment, and frailty cannot be entirely excluded. Nonetheless, a comprehensive meta-analysis pooling over 651,000 patients confirmed that baseline thrombocytopenia was independently associated with increased in-hospital mortality and bleeding after PCI, even after adjustment for confounders [15], supporting the validity of our adjusted analyses. Future studies employing propensity matching or instrumental variable approaches are warranted to more definitively address this question.

### Mild thrombocytopenia: a clinically manageable condition?

The observation that mild thrombocytopenia (100,000–150,000/mm³) was not significantly associated with adverse outcomes after multivariate adjustment deserves consideration. Several factors may explain this finding. First, although the mild subgroup was the largest (*n* = 237), event rates were relatively low, which may have limited the statistical power to detect small but clinically meaningful differences. Second, mild thrombocytopenia in PCI patients may often be transient, related to haemodilution, medication effects, or post-procedural recovery, and may not carry the same pathological significance as persistent moderate or severe forms. Third, patients with mild thrombocytopenia may have been managed more conservatively (e.g., preferential radial access, shorter DAPT duration), potentially attenuating adverse outcomes, though this was not systematically captured in our study.

These observations are consistent with existing literature. Shiraishi et al. reported that elective PCI appeared feasible in patients with mild thrombocytopenia, with no significant increase in in-hospital mortality compared to those with normal platelet counts [24]. Similarly, Ye et al. demonstrated that PCI in ACS patients with mild-to-moderate thrombocytopenia was associated with improved in-hospital outcomes compared to medical therapy alone, with only minor bleeding risks reaching significance [25]. These findings collectively suggest that mild thrombocytopenia, while warranting monitoring, may not require aggressive modification of standard antiplatelet strategies.

### Bleeding complications and BARC events

Our study identified a pattern in bleeding complications assessed using the BARC criteria, similar to the trend observed for MACE. Patients with mild thrombocytopenia did not show a significant increase in bleeding risk (adjusted HR: 1.02, *p* = 0.80). However, those with moderate thrombocytopenia experienced a significant elevation in bleeding risk (adjusted HR: 1.65, *p* < 0.001), while severe thrombocytopenia demonstrated the highest risk (adjusted HR: 2.70, *p* < 0.001). These findings align with studies showing that thrombocytopenia significantly raises bleeding risks in patients undergoing PCI, with hazard ratios for moderate to severe thrombocytopenia ranging from 2.18 to 3.49 for major bleeding events [14, 26].

The mechanisms driving this increased bleeding risk likely involve reduced platelet aggregation, impaired clot formation, and heightened susceptibility to procedural complications. Another study confirmed that thrombocytopenia independently predicts major bleeding in cardiovascular patients, particularly when combined with antithrombotic therapies [27]. These findings emphasize the need for tailored management strategies to mitigate bleeding risks in patients with moderate to severe thrombocytopenia undergoing invasive procedures [22, 28].

### In-hospital mortality and secondary outcomes

Our study also demonstrated a significant association between thrombocytopenia severity and in-hospital mortality. The overall mortality rate was 4.3%, ranging from 2.1% in the mild thrombocytopenia group to 14.8% in the severe group. Severe thrombocytopenia was a significant predictor of in-hospital mortality, with an adjusted odds ratio (OR) of 4.12 (*p* < 0.001). This observation aligns with research indicating that thrombocytopenia nearly doubles the odds of mortality and increases the risk of other adverse outcomes such as cardiogenic shock and ischemic stroke [16]. However, given the small sample size in the severe subgroup (*n* = 27), the wide confidence intervals (95% CI: 1.90–8.93) and the potential for Type I error or overfitting in multivariable models should be acknowledged; these findings should therefore be considered hypothesis-generating and require validation in larger, adequately powered cohorts.

Secondary outcomes, including stent thrombosis, target vessel revascularization, and post-PCI MI, were also significantly linked to thrombocytopenia severity. Post-PCI MI was observed in 6.2% of patients, with severe thrombocytopenia carrying an adjusted OR of 3.50 (*p* < 0.001). Research supports that acquired thrombocytopenia after PCI is associated with increased rates of both thrombotic and hemorrhagic complications, highlighting the complex interplay between coagulation abnormalities and vascular injury [15].

### Subgroup analysis: risk factors and comorbidities

Our subgroup analysis revealed that the severity of thrombocytopenia was significantly associated with certain patient characteristics. Notably, smoking was more prevalent among patients with moderate thrombocytopenia (72.5%, *p* = 0.0065), and increased smoking intensity correlated with greater severity of thrombocytopenia. The biological plausibility for this association is supported by robust evidence. Ishida et al. comprehensively demonstrated that cigarette smoking induces chronic endothelial dysfunction, systemic inflammation via NLRP3 inflammasome and cGAS-STING pathway activation, enhanced platelet aggregation and consumption, and altered coagulation cascade balance [29]. Although smoking typically increases platelet reactivity, this heightened activation and consumptive coagulopathy may paradoxically contribute to lower circulating platelet counts in the setting of chronic inflammation. Ząbczyk et al. further showed that smoking promotes the formation of dense, lysis-resistant fibrin clots and adversely modifies platelet adhesion through upregulation of adhesion molecules and enhanced oxidative stress, contributing to a prothrombotic milieu that can deplete circulating platelets [30]. This finding aligns with studies demonstrating that smoking exacerbates cardiovascular risks through pro-inflammatory and pro-thrombotic mechanisms, which may worsen thrombocytopenia-related outcomes [31].

Additionally, a history of prior PCI and MI emerged as significant predictors of severe thrombocytopenia, with adjusted odds ratios (ORs) of 2.76 and 2.33, respectively. This association likely reflects the cumulative impact of antiplatelet therapy exposure, repeated procedural platelet consumption, and the underlying chronic inflammatory burden associated with coronary artery disease. Park et al. reported that baseline thrombocytopenia in patients undergoing PCI with drug-eluting stents was significantly associated with older age, more comorbidities, and higher cardiovascular risk profiles, suggesting that thrombocytopenia in this population is a marker of cumulative disease burden [32]. Similar associations have been reported in studies indicating that patients with a history of cardiac interventions or ACS are at elevated risk of thrombocytopenia-related adverse outcomes [33]. Furthermore, hypertension and dyslipidemia were more prevalent in patients with moderate and severe thrombocytopenia, emphasizing the multifactorial nature of cardiovascular risk in these individuals [32].

### Lipid profiles and cardiovascular risk

Although lipid profiles did not show statistically significant differences across thrombocytopenia grades, certain trends were evident. Patients with severe thrombocytopenia exhibited lower HDL cholesterol levels (mean: 35.75 mg/dL), which could have implications for cardiovascular risk. However, total cholesterol and LDL cholesterol levels were not significantly associated with thrombocytopenia severity. Research on cardiovascular patients with thrombocytopenia has similarly reported inconsistent findings on lipid profiles, suggesting the need for further investigation to clarify these associations [34].

### Antithrombotic strategies in thrombocytopenic patients

The management of antithrombotic therapy in thrombocytopenic patients undergoing PCI is particularly challenging. In our study, DAPT consisted of aspirin combined with a P2Y12 inhibitor — clopidogrel in 86.1%, ticagrelor in 13.0%, and prasugrel in 0.8% of patients. DAPT duration was generally maintained for 6–12 months per institutional protocol; however, no formal de-escalation strategy was prospectively applied.

Emerging evidence strongly supports abbreviated DAPT regimens in high-bleeding-risk patients. In the STOPDAPT-2 trial, Watanabe et al. demonstrated that 1-month DAPT followed by clopidogrel monotherapy significantly reduced major bleeding in high-bleeding-risk patients (defined by ARC-HBR criteria) compared to 12-month DAPT, with a numerically greater bleeding reduction benefit in the HBR subgroup [35]. Similarly, in the landmark TWILIGHT trial, Mehran et al. showed that ticagrelor monotherapy after 3 months of DAPT reduced clinically relevant bleeding (BARC 2, 3, or 5) by 44% compared to continued ticagrelor plus aspirin, without increasing ischemic events among high-risk PCI patients [36]. These findings are directly relevant to our thrombocytopenic cohort, where the balance between ischemic protection and bleeding avoidance is critical.

Furthermore, P2Y12 inhibitor monotherapy is emerging as a promising bleeding mitigation strategy. Oliva et al. conducted a meta-analysis of five randomized trials including 31,627 patients and demonstrated that P2Y12 inhibitor monotherapy after a short course of DAPT (1–3 months) significantly reduced major bleeding compared to standard DAPT (≥ 12 months), with a consistent treatment effect in both complex and non-complex PCI patients [37]. Notably, in patients undergoing complex PCI — a population with both high ischemic and high bleeding risk — P2Y12 monotherapy was associated with a reduced risk of MI (HR: 0.77, 95% CI: 0.60–0.99) compared to standard DAPT, suggesting that early aspirin withdrawal may offer net clinical benefit rather than harm. These findings support the consideration of transitioning to P2Y12 monotherapy after a brief DAPT period in thrombocytopenic patients to optimize the balance between ischemic protection and bleeding avoidance.

### Patients with atrial fibrillation: a uniquely high-risk subgroup

A notable proportion of patients undergoing PCI in clinical practice have concomitant AF, requiring oral anticoagulation in addition to antiplatelet therapy. In our cohort, no patients (0/368) had AF at baseline. This absence is attributable to our exclusion criteria, which excluded patients on oral anticoagulants to isolate the effect of DAPT on outcomes without the confounding influence of concomitant anticoagulation. While this design choice improved internal validity, it limits the generalizability of our findings to the substantial real-world population of thrombocytopenic patients with coexisting AF.

Patients with AF undergoing PCI represent a uniquely high-risk population, as the overlap of oral anticoagulation with DAPT (i.e., triple antithrombotic therapy) substantially increases bleeding risk, particularly in the setting of thrombocytopenia. Castiello et al. recently provided an excellent review of procedural and antithrombotic therapy optimization in patients with AF undergoing PCI, emphasizing that dual antithrombotic therapy (an oral anticoagulant plus a single antiplatelet agent) has emerged as the preferred strategy over triple therapy for reducing bleeding without a significant increase in ischemic events [38]. In patients with coexisting thrombocytopenia, the additional haemorrhagic burden of overlapping anticoagulation and antiplatelet agents would be expected to be particularly harmful, making careful antithrombotic strategy selection even more critical. Future studies should specifically include thrombocytopenic patients with AF to evaluate optimal antithrombotic management in this high-risk overlap population, which was not captured by our study design.

### Integration of bleeding risk scores

The practical applicability of our findings could be enhanced by integrating validated bleeding risk assessment tools. The PRECISE-DAPT score, developed and validated by Costa et al. using pooled data from over 14,000 patients across eight randomized trials, is a five-item score (age, creatinine clearance, haemoglobin, white blood cell count, and prior spontaneous bleeding) that demonstrated a c-index of 0.73 for predicting out-of-hospital TIMI major/minor bleeding during DAPT [11]. Importantly, patients scoring ≥ 25 derived no net benefit from prolonged DAPT, whereas those with lower scores had reduced ischemic events with longer treatment. The ARC-HBR criteria, validated by Ueki et al. in over 12,000 PCI patients, showed higher sensitivity for bleeding prediction (63.8%) compared to other contemporary risk scores and identified that 39.4% of PCI patients met HBR criteria [12]. Notably, thrombocytopenia is incorporated as a recognized risk factor within the ARC-HBR framework.

We recommend that clinicians integrate these validated scoring tools with thrombocytopenia severity grading to enable more nuanced clinical decision-making regarding DAPT duration and intensity. The combination of thrombocytopenia severity with PRECISE-DAPT or ARC-HBR scores may provide a more comprehensive risk profile than either approach alone.

### Clinical implications

The findings from this study underscore the importance of early risk stratification in patients with thrombocytopenia undergoing cardiovascular interventions. Identifying patients at higher risk for MACE, bleeding complications, and in-hospital mortality is critical to optimizing treatment strategies. However, it is important to emphasize that our study did not directly test alternative management strategies, and therefore specific therapeutic recommendations should be interpreted with caution. Tools that incorporate thrombocytopenia severity into risk assessment models, such as the PRECISE-DAPT score and ARC-HBR criteria, may improve clinical decision-making, particularly concerning the use of antiplatelet and anticoagulant therapies. This aligns with current research advocating for tailored approaches to managing both thrombotic and hemorrhagic risks in thrombocytopenic patients [39].

Furthermore, our results highlight the need for multidisciplinary care strategies that address both hematologic and cardiovascular risk factors. Future studies should explore targeted interventions, including the role of abbreviated DAPT regimens, P2Y12 monotherapy, and novel antithrombotic agents, to mitigate these risks in prospective, randomized settings [40].

### Limitations

Several limitations of this study should be acknowledged. First, as a single-centre study conducted at a tertiary care hospital in Northern India, the findings may not be generalizable to other healthcare settings, geographic regions, or ethnic populations. The Indian population may have unique epidemiological profiles, including different prevalences of comorbidities (e.g., diabetes, rheumatic heart disease), varying access to newer-generation stents and P2Y12 inhibitors, and distinct genetic determinants of platelet function and antiplatelet drug metabolism (e.g., CYP2C19 polymorphisms). Antiplatelet prescribing practices and procedural techniques (access site preferences, stent selection) may also differ across institutions and countries. Multi-centre, multi-ethnic studies are essential to confirm and extend these results.

Second, the severe thrombocytopenia subgroup comprised only 27 patients, which may limit statistical power, increase the risk of Type I error, and potentially lead to overestimation of effect sizes in multivariable models. A post-hoc power analysis indicated that with this sample size, the study had limited power (~ 50–60%) to detect moderate effect sizes in the severe subgroup, and the wide confidence intervals observed (e.g., HR: 2.10, 95% CI: 1.72–2.58 for MACE; OR: 4.12, 95% CI: 1.90–8.93 for in-hospital mortality) reflect this uncertainty. These findings in the severe subgroup should therefore be considered hypothesis-generating and require validation in larger, adequately powered cohorts.

Third, the follow-up period was limited to 6 months, which may not capture late ischemic events such as very late stent thrombosis (which can occur beyond 1 year, particularly with drug-eluting stents), late target vessel revascularization, or delayed bleeding complications that may emerge during prolonged antiplatelet therapy. Given that some of the most clinically significant adverse events in PCI patients — including neoatherosclerosis-related stent failure and late bleeding from chronic medication use — occur beyond the 6-month window, longer-term follow-up studies (≥ 12–24 months) are recommended to provide a comprehensive assessment of outcomes in thrombocytopenic patients.

Fourth, despite multivariate adjustments, the observational nature of the study leaves room for unmeasured confounding variables. Specifically, frailty indices were not captured, and frailty is increasingly recognized as an independent predictor of adverse outcomes following PCI. Medication adherence, which is known to vary substantially in real-world settings and directly affects both ischemic and bleeding outcomes, was not monitored. Procedural complexity variables — including the number of stents deployed, lesion length, bifurcation involvement, use of intravascular imaging, and vascular access site (radial vs. femoral) — were not systematically incorporated into our regression models. Concurrent medications beyond DAPT, including proton pump inhibitors, non-steroidal anti-inflammatory drugs, and herbal supplements that may affect platelet function, were also not fully accounted for. These unmeasured variables may have influenced the observed associations and should be addressed in future prospective studies.

Fifth, DAPT duration was not standardized in our cohort and was largely determined by individual clinician judgment; we were therefore unable to stratify outcomes by treatment length, which is a recognized modifier of both bleeding and ischemic risk. Given the strong evidence from the STOPDAPT-2 [35] and TWILIGHT [36] trials that DAPT duration modifies adverse outcomes, future prospective studies should formally incorporate DAPT duration as a stratification variable.

Sixth, propensity score matching was not performed due to the modest sample size, and residual confounding from liver disease, renal impairment, malignancy, and other systemic conditions contributing to thrombocytopenia cannot be entirely excluded.

Finally, patients with concomitant AF requiring oral anticoagulation were excluded from the study, resulting in no AF patients (0/368) in the cohort. While this exclusion improved the internal validity of assessing DAPT-related outcomes in isolation, it precludes any analysis of the important clinical scenario where thrombocytopenic patients require combined anticoagulant and antiplatelet therapy — a population at particularly elevated bleeding risk that warrants dedicated investigation.

## Conclusion

This study demonstrates a graded association between thrombocytopenia severity and adverse outcomes in patients undergoing PCI while on DAPT. Moderate and severe thrombocytopenia were independently associated with higher risks of bleeding complications and MACE. Severe thrombocytopenia, in particular, was associated with a markedly increased risk of in-hospital mortality, although the small subgroup size (*n* = 27) and wide confidence intervals warrant cautious interpretation, and these findings should be considered hypothesis-generating pending validation in larger cohorts.

Our observations are consistent with existing literature showing that thrombocytopenia significantly exacerbates bleeding risks during DAPT. While we did not directly evaluate alternative treatment strategies, emerging evidence from trials such as STOPDAPT-2 and TWILIGHT suggests that abbreviated DAPT durations and early de-escalation to P2Y12 inhibitor monotherapy may offer promising approaches for reducing bleeding without compromising ischemic outcomes in high-bleeding-risk patients. The integration of validated risk assessment tools, including the PRECISE-DAPT score and ARC-HBR criteria, alongside thrombocytopenia severity grading, may assist clinicians in making more individualized DAPT decisions.

Several important limitations must be acknowledged, including the single-centre design, small severe subgroup, short follow-up duration (6 months), non-standardized DAPT duration, and potential for residual confounding from unmeasured variables. Future multi-centre studies with larger sample sizes, longer follow-up periods, and prospective evaluation of modified antiplatelet regimens in thrombocytopenic patients are needed to translate these observational findings into evidence-based management recommendations.

## Data Availability

No datasets were generated or analysed during the current study.

## Abbreviations

CVDs: Cardiovascular diseases
ACS: Acute Coronary syndrome
PCI: Percutaneous coronary intervention
DAPT: Dual-antiplatelet therapy
MI: Myocardial infarction
MACE: Major adverse cardiovascular events
BMS: Bare-metal stents
DES: Drug-eluting stents
BARC: Bleeding academic research consortium
SD: Standard deviation
ROC: Receiver operating characteristic
CI: Confidence interval
HR: Hazard ratio
OR: Odds ratio
SMS: Sawai man Singh (Medical College)
SPSS: Statistical package for the social sciences
BMI: Body mass index
HDL: High-Density lipoprotein
LDL: Low-density lipoprotein
AF: Atrial fibrillation
ARC-HBR: Academic research consortium for high bleeding risk
HBR: High bleeding risk
SAPT: Single antiplatelet therapy
